# Assessing physicians’ training needs in anatomic pathology requests and specimen handling: An FSP grid analysis from a Moroccan university hospital

**DOI:** 10.1016/j.acpath.2026.100278

**Published:** 2026-07-07

**Authors:** Kenza Oqbani, Salma Akkari, Sanae Abbaoui, Mounsif Birouk, Najia Hajjaj-Hassouni

**Affiliations:** a Laboratory of Biotechnologies and Medicine, Faculty of Medicine and Pharmacy, Ibn Zohr University, Agadir, Morocco; b Sheikh Khalifa International University Hospital, Mohammed VI University of Sciences and Health, Casablanca, Morocco; c Traumatology and Orthopedic Surgery, Private Medical Practice, Casablanca, Morocco; d Faculty of Medicine and Pharmacy, Mohammed V University, Rabat, Morocco

**Keywords:** Continuing medical education, Frequency-severity-problems grid, Pathology request forms, Pre-analytical errors, Resource-limited settings, Sample management

## Abstract

Incomplete or noncompliant pathology request forms are a major source of pre-analytical errors worldwide, compromising diagnostic accuracy and delaying patient care. Evidence from low-resource settings like North Africa remains scarce. This first study in Morocco systematically assessed physicians’ training needs in pathology request completion and specimen handling, using the validated Frequency-Severity-Problems grid to prioritize continuing medical education. A mixed-methods training-needs assessment was conducted with 24 medical interns at a university hospital in northwestern Morocco using individual interviews and a questionnaire. It focused on three domains: pathology request form completion, specimen vial labeling, and formalin fixation. Quantitative prioritization of training needs used the Frequency-Severity-Problems grid. All interns reported unmet training needs. Three critical deficiencies were identified: incomplete pathology request forms (75% lacked key clinical data), inaccurate vial labeling (58% reported errors), and inadequate knowledge of fixation procedures (62%). Mean Frequency-Severity-Problems scores confirmed these as training priorities: 4.95 for form completion, 2.08 for labeling, and 3.41 for fixation. Deficits involved knowledge, technical skills, and attitudes. Significant pre-analytical gaps exist among medical interns. Targeted continuing medical education on standardized pathology requests is urgently needed to reduce diagnostic errors and enhance patient outcomes in low-resource settings.

## Introduction

Pathologists play a central role in diagnosing, staging, and guiding the treatment and follow-up of cancers and numerous other diseases. Accurate pathological diagnosis relies on high-quality specimens and appropriate anatomoclinical correlation, both of which depend on complete and relevant clinical information transmitted through pathology request forms.^1^ International guidelines emphasize the need to include adequate clinical context, specimen origin, pre-operative findings, and suspected diagnoses to ensure optimal sample processing, appropriate ancillary testing, and accurate interpretation.^1^^,^^2^

According to Benoît et al., the quality of communication between clinicians and pathologists is a cornerstone of effective cancer care.^3^ Nevertheless, in daily practice, pathology laboratories often receive incomplete, inaccurate, or unsigned request forms, incorrect or missing labeling, and inadequately fixed specimens. These nonconformities have significant implications: increased pre-analytical errors, delayed reporting, unnecessary laboratory workload, and potential diagnostic inaccuracies.^4^ Previous studies have documented that most laboratory errors originate in the pre-analytical phase.

In Morocco, no published data have examined pre-analytical challenges or physicians’ training needs related to pathology request preparation and specimen handling. This study addresses this gap by assessing medical interns’ competencies and training needs using the validated Frequency-Severity-Problems (FSP) grid. By identifying deficiencies in knowledge, technical skills, and attitudes, this research provides actionable insights to improve pathology request quality and specimen management.

## Materials and methods

### Ethical considerations

An anonymous questionnaire was administered to medical interns who voluntarily participated in this survey-based study. No personal, identifiable, or sensitive data were collected. In line with institutional and national ethical guidelines, this minimal-risk study is exempt from formal ethics committee review. All participants were informed about the study’s purpose, voluntary nature, and anonymity of responses. Completing the questionnaire implied informed consent. All procedures adhered to the 1964 Declaration of Helsinki and its later amendments.

### Study design and population

**Fig. 1:**
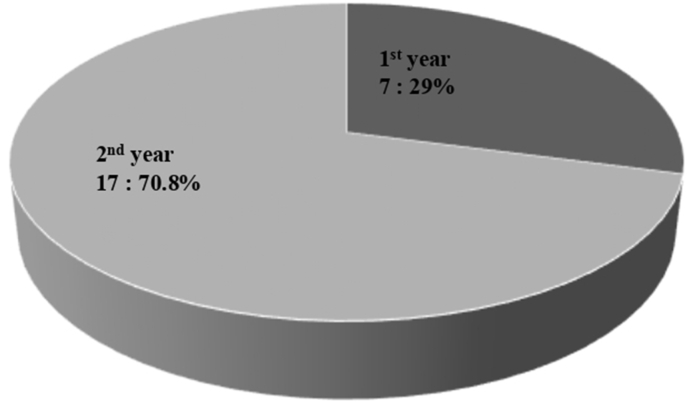
Sample distribution according to the year of medical internship.

This cross-sectional mixed-methods study was conducted among 24 medical interns from various clinical and surgical departments at a university hospital in northwestern Morocco (Fig. 1). Participants were those assigned to departments involved in sending specimens to the pathology laboratory and who were available on the day of data collection. All eligible interns present agreed to participate and completed both the interviews and the questionnaire.

In the Moroccan medical training system, interns are junior physicians in the early postgraduate phase typically having less than two years of clinical experience following graduation. As a result, the sample was relatively homogeneous in terms of clinical experience, limiting variability in this parameter. Importantly, these medical interns are the primary physicians responsible for completing pathology request forms and handling specimens in the university hospital setting, making their level of training particularly relevant when interpreting the identified gaps.

All participants had previously submitted specimens to the pathology laboratory (Table 1) and represent a substantial proportion of physicians involved in completing pathology request forms and labeling patient specimens.

**Table 1:** Sample distribution according to the current department/service.

Actual service/department	N	Percentage (%)
Traumatology	2	8.3
Neurosurgery	3	12.5
Pediatrics	2	8.3
Urology	2	8.3
Pneumology	1	4.1
Internal medicine	2	8.3
Obstetrics-gynecology	1	4.1
Cardiology	1	4.1
Ear, nose, and throat	1	4.1
Nephrology	1	4.1
General surgery	1	4.1
Dermatology	1	4.1
Radiology	4	12.5
Vascular Surgery	1	4.1
Anesthesia and intensive care	1	4.1

N: Number.

### Data collection

*Mixed-methods data collection*. This study employed a mixed-methods approach combining qualitative and quantitative data collection to comprehensively assess training needs. The needs analysis approach was based on two complementary tools: semi-structured individual interviews, and an anonymous questionnaire incorporating open-ended items. Collected responses were subsequently analyzed and prioritized using the validated FSP grid, enabling the identification of key gaps across three domains: knowledge, technical skills (know-how), and attitudes (soft skills).

1. Individual Interviews: Qualitative component

Semi-structured individual interviews were conducted with all participants to explore their perceived difficulties and training needs related to anatomic pathology practices. The interviews aimed to assess interns’ understanding of the importance of anatomic-clinical correlation and their ability to provide relevant clinical information to pathologists. A flexible interview guide was used, focusing on three main thematic areas^1^: completion of pathology request forms, including the type and relevance of clinical information provided^2^; specimen vial labeling practices; and^3^ knowledge and practices related to specimen fixation, particularly the use of formalin. Interviews allowed participants to freely describe challenges encountered in daily practice and to express their educational needs.

2. Questionnaire and FSP grid: Quantitative component

A structured questionnaire was administered to all participants to quantify the identified training needs detected during interviews. It covered the same three domains: pathology request form completion, vial labeling, and immediate placement of specimens in formalin.

The training needs questionnaire was assessed using the FSP grid, a published validated tool proposed by d'Ivernois JF,^5^ for prioritizing continuing medical education objectives.

For each domain, participants rated as follows: frequency (F) of occurrence (0 = rare, 1 = moderate, and 2 = frequent), severity (S) of consequences (0 = low, 1 = moderate, and 2 = high), and problems (P) encountered (0 = none, 2 = moderate, and 4 = significant) based on their personal experience. The “Problems” dimension was further explored across three subdomains: knowledge, technical skills (know-how), and attitudes (soft skills). Scores were aggregated to generate total and mean values for each item, allowing prioritization of training needs based on their combined frequency, severity, and impact on practice.

The FSP grid applied in this study (Table 2) was adapted from the framework described by d’Ivernois and customized by the authors for the purposes of this study.

**Table 2:** FSP grid used to assess training needs in continuing medical education (according to d’Ivernois JF^5^).

Items	Frequency	Severity	Problems			Total
			Knowledge	Know-how	Soft skills	
Preparation of the pathological examination request	0 1 2	0 1 2	0 2 4	0 2 4	0 2 4	
Vial labeling	0 1 2	0 1 2	0 2 4	0 2 4	0 2 4	
Immediate sample placement in formalin	0 1 2	0 1 2	0 2 4	0 2 4	0 2 4	
*Frequency (F): rated as 0, 1, or 2 based on the participant’s assessment of its occurrence in their professional practice.* • *0: rare* • *1: moderately frequent* • *2: very frequent* *Severity (S): similarly rated as 0, 1, or 2, based on the participant’s assessment of its severity in their professional practice.* • *0: benign* • *1: moderately severe* • *2: very severe* *Problems (P): The scoring process follows the same approach as previously, but here, the problems are rated 0, 2, or 4. This scoring helps highlight the problems and thus the training needs in CME, in relation to the concepts of frequency and severity.These can include issues related to knowledge, manual skills (know-how), or interpersonal skills (soft skills or knowledge being).* • *0: no problem* • *2: moderate problems* • *4: significant problems*						

FSP: Frequency-Severity-Problems; N: Number.

## Results

Individual interviews with all medical interns revealed perceived difficulties in submitting histopathological examination requests and a unanimous need for continuing medical education (CME). All participants (100%) recognized the necessity of anatomoclinical confrontation for a comprehensive diagnosis approach.

Three relevant key issues emerged from the individual interviews and were quantified using an FSP analysis grid (Table 3, Table 4). These issues concerned the need for a well-completed, dated, and signed request form, proper labeling of specimen vials, and the immediate placement of samples in formalin.

**Table 3:** Example of the FSP grid results provided by a questionnaire participant.

Items	Frequency N	Severity N	Problems			Total N
			Knowledge N	Know-how N	Soft skills N	
Preparing the pathology examination request	1	1	2	2	2	8
Vial labeling	2	2	2	2	2	8
Immediate sample placement in formalin	2	2	2	2	2	10

FSP: Frequency-Severity-Problems; N: Number.

**Table 4:** FSP grid, including total values and averages of the scores obtained by item for all participants.

Items	Frequency N (Means)	Severity N (Means)	Problems			Total values of all participants N (Means)
			Knowledge N (Means)	Know-how N (Means)	Soft skills N (Means)	
Preparation of the pathology examination request	19 (0.79)	27 (1.12)	18 (0.75)	31 (1.29)	24 (1)	119 (4.95)
Vial labeling	18 (0.75)	13 (0.54)	4 (0.16)	8 (0.33)	7 (0.29)	50 (2.08)
Immediate sample placement in formalin	11 (0.45)	18 (0.75)	19 (0.79)	19 (0.79)	15 (0.62)	82 (3.41)

FSP: Frequency-Severity-Problems; N: Number. The scores result from the application of the FSP grid (d’Ivernois JF^5^).

Quantitative analysis confirmed these as critical training needs. The total mean scores for difficulties were 4.95 for request forms, 2.08 for vial labeling, and 3.41 for formalin fixation (see Table 4).

A detailed breakdown, illustrated in Table 3, showed that request form deficiencies scored highest overall, particularly in the severity of the issue and a lack of knowledge. In contrast, vial labeling was identified as a less severe but frequently occurring problem. Furthermore, failures in correct fixation scored highly for both their severe consequences and a distinct lack of technical competence, or know-how, among the participants.

Participants frequently reported uncertainty about which clinical details to include, how to properly label specimens, and the correct volume and timing of formalin fixation.

## Discussion

In routine practice, pathology laboratories receive a significant number of specimens daily for pathological examination, and pre-analytical noncompliance is widely reported in the literature.^6^ These include incomplete and/or unsigned request forms lacking clinical data, as well as inadequate specimen handling. These issues—such as mislabeling and delayed fixation, especially on weekends or during public holidays—compromise tissue integrity through autolysis and degrade diagnostic quality.^7^^,^^8^ Our survey identified critical training gaps among medical interns, the future physician workforce, specifically in completing histopathology requests, labeling vials, and proper formalin fixation. Interns reported struggles in determining essential clinical data for pathologists and expressed deficits in knowledge, technical skills (know-how), and professional attitudes across all three areas.

This problem is widespread. Previous studies confirm that suboptimal request forms are common.^9^ Errors during the pre-analytical phase, which occur prior to the specimen reaching the pathology laboratory, account for the majority of laboratory mistakes with some studies reporting up to 10% of specimens arriving without any request form.^10^ A significant proportion of issues in surgical pathology specimen identification and processing stem from missing or incorrect clinical information.^11^ A major contributor is insufficient information from clinicians^12^^,^^13^ with pre-analytical errors accounting for 60–77% of all laboratory mistakes.^14^ These errors often stem from clinicians underestimating their crucial role in the pre-analytical phase of diagnostic procedures.^12^^,^^13^

International guidelines and the ISO 15189:2007 standard underscore that proper requisition is fundamental for quality assurance in pathology laboratories.^1^^,^^7^^,^^15^ These standards define the pre-analytical phase as beginning with the clinician’s request, encompassing examination requisition, patient preparation, sample collection, and transportation to the laboratory, concluding when the analytical phase begins.^7^ According to these international protocols, all specimens must be accompanied by request forms including essential clinical information to ensure optimal sample management and support accurate pathological reporting.^1^^,^^15^ A properly completed pathology request form thus serves as a crucial component in quality control, streamlining effective sample management and enabling the production of reliable diagnostic reports.^16^

The consequences are substantial. Noncompliances create additional work for pathologists, who must track down missing data or modify specimen processing, leading to unnecessary sections and investigations. More critically, the lack of crucial information can result in misinterpretation and diagnostic errors, severely impacting patient care.^12^ Besides these issues, the completeness of clinical data—such as medical history and differential diagnosis—on request forms can affect the turnaround time of anatomic pathology examinations and their timely analysis.^17^

Our findings highlight a lack of awareness among interns about the request form’s role as a vital diagnostic tool. To address these challenges, we propose a multi-faceted approach: creating updated standard request forms with requesting clinician input, establishing clear protocols for pathology staff to return noncompliant samples, especially when clinical data are critical for accurate analysis. Most importantly, organizing targeted educational sessions to emphasize the importance of pre-analytical quality remains the key strategy.

Deficiencies in anatomic pathology request forms represent a critical pre-analytical challenge, contributing to diagnostic inaccuracies and workflow inefficiencies in histopathology laboratories. While prior studies have characterized these errors in high-income settings, there is a paucity of data from low- and middle-income countries, where resource constraints may exacerbate the problem. To our knowledge, this represents the first systematic assessment of pathology request training gaps among medical interns in Morocco. Our study introduces the novel application of the FSP grid-a validated tool for prioritizing CME objectives-to quantify pre-analytical errors in anatomic pathology.

### Limitations

This study has limitations, including a small sample size confined to medical interns at a single center, which reduces the generalizability of our findings. A broader survey involving residents, general practitioners, and specialists would be more representative and would have enabled a comparative and correlational study. Future multicenter studies with larger cohorts and additional assessment tools are needed to validate these findings and assess their broader applicability.

## Conclusion

Accurate pathology diagnosis depends heavily on the quality of pre-analytical processes. This study demonstrates substantial training gaps among future Moroccan physicians in completing pathology request forms and handling specimens. Strengthening CME programs, standardizing request forms, and reinforcing communication with pathology departments are key strategies to reduce errors and improve diagnostic accuracy in resource-limited settings.

Clinicians are encouraged to provide complete and detailed clinical information to support timely and precise histopathological diagnosis, ultimately improving patient outcomes. All relevant clinical details should be included in the request forms, thereby fostering a clearer pathway to multidisciplinary management, precise diagnoses, and better clinical outcomes.

## Author contributions

O.K. conceived the idea was involved in the study’s conception and design, material preparation, data acquisition and interpretation, and manuscript drafting. Data collection, including individual interviews and questionnaire distribution, was conducted by S.A. M.B was involved in the statistical evaluation of data and helped in drafting the article. The author H–H.N. was involved in the study approval and reviewed and approved the final version of the manuscript. All authors read and approved the final manuscript.

## Disclaimer

The views expressed in this article are those of the authors and are the product of professional research and do not necessarily represent the positions of affiliated institutions or the publisher. The authors are responsible for this article’s results, findings, and content.

## Funding

This research received no specific grant from any funding agency in the public, commercial, or not-for-profit sectors.

## Declaration of competing interest

On behalf of all authors, the corresponding author states that there is no conflict of interest.
